# Development of an Automated Low-Cost Multispectral Imaging System to Quantify Canopy Size and Pigmentation

**DOI:** 10.3390/s24175515

**Published:** 2024-08-26

**Authors:** Kahlin Wacker, Changhyeon Kim, Marc W. van Iersel, Benjamin Sidore, Tony Pham, Mark Haidekker, Lynne Seymour, Rhuanito Soranz Ferrarezi

**Affiliations:** 1 Department of Horticulture, University of Georgia, Athens, GA 30602, USA; kahlin.wacker@uga.edu (K.W.); mvanier@uga.edu (M.W.v.I.); benjaminsidore@gmail.com (B.S.); 2 Department of Plant Science and Landscape Architecture, University of Connecticut, Storrs, CT 06269, USA; changhyeon.kim@uconn.edu; 3 College of Engineering, University of Georgia, Athens, GA 30602, USA; tmp52468@uga.edu (T.P.); mhaidekk@uga.edu (M.H.); 4 Department of Statistics, University of Georgia, Athens, GA 30602, USA; seymour@uga.edu

**Keywords:** plant image segmentation, anthocyanin, chlorophyll fluorescence, normalized difference vegetation index, normalized difference anthocyanin index

## Abstract

Canopy imaging offers a non-destructive, efficient way to objectively measure canopy size, detect stress symptoms, and assess pigment concentrations. While it is faster and easier than traditional destructive methods, manual image analysis, including segmentation and evaluation, can be time-consuming. To make imaging more widely accessible, it’s essential to reduce the cost of imaging systems and automate the analysis process. We developed a low-cost imaging system with automated analysis using an embedded microcomputer equipped with a monochrome camera and a filter for a total hardware cost of ~USD 500. Our imaging system takes images under blue, green, red, and infrared light, as well as chlorophyll fluorescence. The system uses a Python-based program to collect and analyze images automatically. The multi-spectral imaging system separates plants from the background using a chlorophyll fluorescence image, which is also used to quantify canopy size. The system then generates normalized difference vegetation index (NDVI, “greenness”) images and histograms, providing quantitative, spatially resolved information. We verified that these indices correlate with leaf chlorophyll content and can easily add other indices by installing light sources with the desired spectrums. The low cost of the system can make this imaging technology widely available.

## 1. Introduction

Imaging is a projection of the interaction between light and objects. Image sensors or cameras capture reflected light from objects and convert the intensity of the reflection into signals. These signals are stored as pixels that consist of intensity and spatial information. Following the principles of imaging, capturing an image of a plant records its reflectance as pixels and their spatial data, while differences in pixel intensity depict the variation in reflectance. At the same time, changes in reflectance in plants can be used to detect changes in plant physiological status and growth [1,2]. Thus, plant reflectance as measured by image sensors has the potential capability to measure canopy size, yield, disease infection, and symptoms related to physiological disorders as well as phenotypical data, as plant materials have shown unique spectral responses at various wavelengths, associated with differences in pixel intensity [3].

Image-derived reflectance can be used to calculate widely used indices such as the normalized difference vegetation index (NDVI) [4] and the photochemical reflectance index (PRI) [5] and detect parameters of interest and phenotypes combined with various machine learning algorithms using pixel intensity as an independent variable [6]. Reflectance-based phenotyping is not a recent technology, because satellite imagery [4] or direct reflectance measurement [7] has been used to estimate the phenotypic variation of plants. Advances in technology have provided both low-cost, high-resolution cameras and more affordable computing power in the commercial market, overcoming previous cost barriers. These aspects of image-based plant analysis allow for plant phenotyping in a non-destructive, rapid, and high-throughput manner [8], while such an approach reduces sample bias [9]. Because of this, image-based plant analysis has experienced increasing interest over the past decade [10,11].

Many research institutes from academia and industry have established fully automated phenotyping centers to identify traits of interest for screening an ideal accession. The decreasing cost, paired with the increasing technological capability, has produced the opportunity for the development of useful research tools. Although there has been outstanding progress in algorithms for plant image analysis with machine learning tools [12], there are still challenges in adopting such a system into controlled environment agriculture (CEA) to monitor plants and potentially control environmental conditions due to cost and complexity. There are a few studies that have shown the possibility of image-based plant analysis to measure plant growth parameters such as canopy size and stem elongation in CEA [13,14,15,16]. However, the cost of these systems has not been realistic to allow growers to install image-based sensors to assist their decision-making in CEA.

Compared to phenotyping in the field or in breeding programs, the application of image-based sensors to monitor plant growth is limited. In particular, adopting image sensors in CEA to facilitate environmental control systems has received less attention than use in fields under the guise of remote sensing. It is well known that growth parameters measured by imaging sensors are useful to achieve an accurate yield prediction in a way traditional sensors cannot offer [15]. However, the cost of such systems was not approachable until recently, and the purpose of the systems was focused on selecting novel traits to facilitate breeding processes. Requirements for processing image sets include computing power and proper analysis methodologies in the code/software. Due to these reasons, the application of imaging sensors to detect plant response has been restricted to usage in phenotyping for novel trait selections.

Open-source hardware and software can reduce the cost of image-based phenotyping systems to facilitate environmental control strategies in CEA. Embedded micro-computers have been used in many research and industry projects due to their accessible and affordable computing power and ease of implementation. Plant scientists also have observed the potential usage of embedded computers to measure crop traits [17]. There are many open-source compatible hardware components and simple coding that allow for a multiplicity of uses for these microcomputers [18]. One benefit is that indoor production in CEA has easier implementation and installation of relevant hardware and allows for reliable reflectance measurements aided by the known spectrum and intensity of the light source, which can be challenging in field conditions.

Many commercial systems exist to measure plant phenotypes in CEA, with varying degrees of adaptability and quality. Commonly available automated phenotyping systems in CEA use a conveyor belt system to move plants through an imaging station. Many commercial imaging systems can be cost-prohibitive for plant scientists and growers in CEA who have limited funding. In addition, many plant scientists and growers in CEA do not initially have the expertise to develop their systems to analyze images in a time-efficient manner. Low-cost systems that automate image collection and analysis can reduce the startup cost in terms of time and money to facilitate the widespread use of image-based sensor technologies to monitor phenotypes of interest. One of the challenges with image analysis is the separation of plant objects from the background [19]. Although many successful cases have achieved accurate plant segmentation using supervised machine learning, implementing such an algorithm may not work when applied to a different cultivar or species or light conditions during image acquisition without retraining the model.

Chlorophyll fluorescence imaging can serve as a robust plant segmentation method by emphasizing the contrast between plants and background objects. Chlorophyll fluorescence imaging provides a simple but effective solution to this problem since it captures fluorescence emitted by chlorophyll in the plant. Upon excitation by actinic light, chlorophyll fluoresces at wavelengths greater than 650 nm [20]. That allows a camera equipped with a long pass filter (>650 nm) and a shorter wavelength light spectrum (usually blue light) to produce higher pixel intensities (brighter) of plant objects in chlorophyll fluorescence images while the background has lower pixel intensity (darker) [21]. Several studies have shown great contrast between plant objects and backgrounds [22,23]. This approach is computationally and physically inexpensive and does not require complicated algorithms for background separation.

Here, we introduce our imaging and image analysis program and a low-cost phenotyping system that takes a chlorophyll fluorescence and multispectral images using an embedded computer to compute and control image collection and analysis. Our objectives were to develop an automated low-cost multi-spectral imaging system that can establish robust plant segmentation, measure plant parameters, and be customized for specific desired outputs, with flexible post-processing. In many research projects, unique theories are conceived, and to allow for testing, an adaptable system must be developed that can be customized to reflect the researcher’s needs. Our goal was to make a system that we could customize to fit our needs, which could be easily changeable for further research into novel methodologies not easily served by off-the-shelf systems.

## 2. Materials and Methods

### 2.1. Image Analysis Program

A program was written in Python (v. 3.8; Python Software Foundation, Wilmington, DE, USA) using the OpenCV library (v. 4.5.4; OpenCV.org, Palo Alto, CA, USA) to process multispectral images. The program and its accompanying instructions are kept as simple as possible to facilitate use by entry-level users with a limited background in programming. The program uses the pixel intensity in images, 0 to 255 in a grayscale image, and a proxy of reflectance at each light condition during imaging, to calculate an index. 

The standalone image analysis program process runs as follows. First, the code reads multispectral images taken under various light colors in a folder and stores them indexed for ease of access. Secondly, the program extracts the pixel intensity and coordinates of each pixel in each spectral image, along with despeckling to reduce noise. Thirdly, the program identifies the chlorophyll fluorescence image, taken under the blue light with a long pass filter (>650 nm), and makes a mask image of plant objects using the chlorophyll fluorescence image, finding a minimum threshold of pixel intensity that separates the background and plant objects and stores it. Fourthly, the code uses the mask image to remove the background whilst retaining plant objects as foreground objects in the spectral image(s) of interest to reduce the processing time by removing the background and allowing for the isolation of valuable parts. The total number of pixels of plant material is summed and converted to total plant area in square centimeters. Fifthly, the code reads the pixel intensity of the spectral image(s) into the index equations and executes the image math calculations to calculate index values for each pixel isolated as the foreground in the mask image phase of operations. Sixthly, the code generates a plant index image and its corresponding histogram quantifying the distribution of pixel intensities in the isolated area of interest, the plant. Finally, in the analysis phase, the code exports the average, standard deviation, and canopy size to a comma-separated values file (.csv) for analysis and comparison. The code then moves to the next folder containing another set of multispectral images and repeats these steps.

From the third step in the analysis code, chlorophyll fluorescence thresholding was referenced. Intensity-based thresholding on chlorophyll fluorescence imaging was used to make a mask image. Chlorophyll fluorescence imaging depicts plant objects and backgrounds differently regarding pixel intensity. Chlorophyll fluorescence imaging results in two peaks: one for the background and another one for plant objects that are photosynthetically active, where the pixels with higher intensity are plant objects. Thus, ignoring pixels below a given intensity using an optimized threshold results in an image only containing plant objects, which will serve as the mask image. A mask image dictates the areas retained and removed from analysis in processing. Since such an optimized threshold value will vary depending on actinic light intensity and the quantity of chlorophyll fluorescence, we added an algorithm to find the optimum value automatically, which finds the minimum value between the two peaks, which is visually displayed in a histogram of the chlorophyll fluorescence image pixel intensity. With the optimized threshold value, the program returns a mask image containing only plant objects, which is then used for foreground imaging in later processes. For the code, maximum and minimum threshold values were set manually, and the code found the optimum value between those points. Direct access to the code is found in the Supplementary Materials.

### 2.2. Validation of Image Analysis Program

To test the image analysis program, multispectral images of leafy vegetables were taken periodically with a commercial multi-spectral imaging system (TopView; Aris, Eindhoven, The Netherlands), which utilizes seven colors of light-emitting diodes (LEDs, 450, 521, 593, 625, 660, 730, and 870 nm) and a long-pass filter (>665 nm) to create a mask image. In the image math calculations, the equation for NDVI, (R_Near Infrared_ − R_Red_)/(R_Near Infrared_ + R_Red_), was used to estimate chlorophyll concentrations, where R is the intensity of the pixel, a measure of reflectance, and the subscript indicates the wavelength.

We compared leaf chlorophyll content measured by portable chlorophyll content meters (CCM-200plus; Opti-Sciences, Hudson, NH, USA) on the plant canopy and canopy NDVI derived from the image analysis program. Red lettuce (*Lactuca sativa*) “Cherokee” red lettuce, green lettuce “Little Gem”, mizuna (*Brassica rapa* var. japonica), and spinach (*Spinacia oleracea*) “Whale F1” were grown in a glass-covered greenhouse in Athens, GA, USA, from 15 June to 13 July 2021. Seeds were planted in 10 cm square pots filled with a soilless substrate (Fafard 2P Mix; Sun Gro Horticulture, Agawam, MA, USA). The average greenhouse conditions were a daily light integral of 23.6 ± 9.7 mol m^−2^ d^−1^, a temperature of 27.0 ± 1.5 °C, and a vapor pressure deficit of 1.3 ± 0.4 kPa (mean ± standard deviation). An 18N-2.6P-10K controlled-release fertilizer (18-6-12 5-6 Month Nursery Mixture; Harrell’s, Lakeland, FL, USA) was added to the pots in different levels (0, 3.75, 5, 7.5, and 10 g of the fertilizer per pot) to induce a wide range of leaf chlorophyll content values in these plants. We prepared five biological replications of each combination of species and fertilizer treatments and selected three homogeneous samples for canopy imaging after 2 weeks post-planting. The leaf chlorophyll content was measured in three representative locations in the plant canopy. The average value of each plant was compared with the canopy NDVI. Nutrient contents of the same plant materials were analyzed and compared with the canopy NDVI.

### 2.3. Low-Cost, Custom System Development

**Figure 1:**
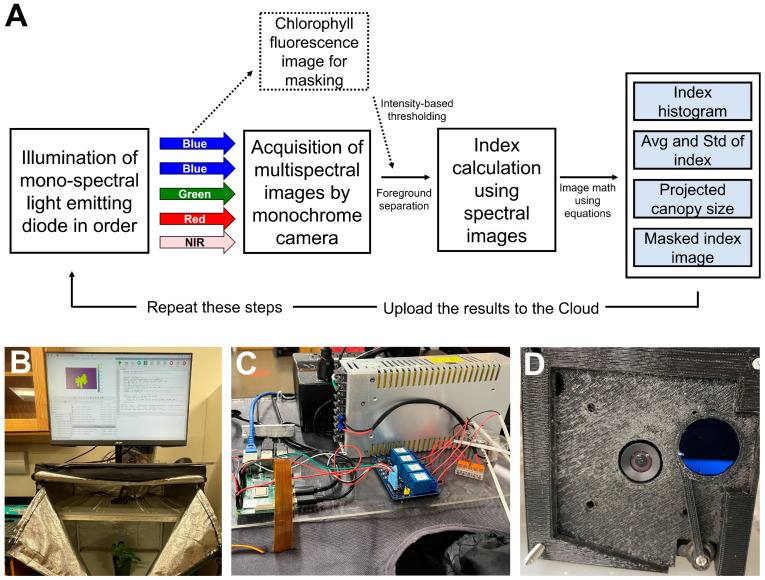
Schematic of the programming logic in the low-cost multispectral imaging system (**A**), overview of low-cost multi-spectral imaging system based on an embedded computer (**B**), wiring and relay array (**C**), and detailed view of chlorophyll fluorescence filter system and bottom view of the camera module (**D**).

Using the commercial system was technically effective, but its optimization for specific applications limited some of the analysis and research needs. Preexisting in the lab was a standalone chlorophyll fluorescence imaging system that lacked the multispectral capabilities desired, and in the process of using the commercial system and conducting the postprocessing of the images, a design was conceived to build a custom multispectral imaging system in-house. A low-cost imaging system prototype, using an embedded microcomputer (Raspberry Pi 4 model B; Raspberry Pi Foundation, Cambridge, UK); a non-IR filtered monochrome camera (OV9281; Arducam, Kowloon, China); blue, green, red, and infra-red LEDs; a movable long-pass filter (LP665; Midwest Optical Systems, Palatine, IL, USA); a generic micro-size hobby servo (MG90S; Maxmoral, China); and assorted custom three-dimensional printed parts, was developed (Figure 1). The microcomputer utilizes the modified image analysis program we developed previously (see Section 2.1), which can run natively in conjunction with the automated multispectral image capture.

The image collection and analysis can be scheduled using a native bash script in the native operating system, and the results can be uploaded to the cloud. The process of image acquisition, processing, and output of the low-cost system is summarized in Figure 1A. This capability was not used in the proof-of-concept studies shown here, but a similar system has been implemented in other studies on-site to monitor plant responses to variations in environmental conditions.

The imaging station was built in a 61 × 61 × 142 cm light-proof grow tent (Mylar Reflective Grow Tent for Indoor Hydroponic Growing; Toolots, Cerritos, CA, USA), which was modified by adding a diffusing acrylic shelf (2 MM High Transmittance Supplier Opal Frosted Cast Milky Double-sizes PS Diffuser Sheet; Sevenneonlighting, Shenzhen, China) near the top (Figure 1B). This was used because light uniformity was a high priority to reduce uneven lighting or brightness-induced anomalies. The lightproof nature of the grow tent is critical for imaging, as the spectrum for each image is provided only by the system and needs to be uninfluenced by supplemental light. LED strips (blue, red, green, and near-infrared) were mounted on top of this shelf, pointed upward to create a diffuse light environment. A monochrome camera (UC-599 Rev. B; ArduCam, China) (Figure 1D) was added, with the lens going through the acrylic diffuser. The camera was calibrated by using a 19 × 25 cm 50% grey card (WhiBal G7; Eclipse Group LLC/Outdoor Photo Gear, Louisville, KY, USA) as a reference. The camera module was enclosed in a three-dimensional printed case [24], which includes a movable long-pass filter (>665 nm) that can be placed in front of the lens. Exposing the plant to blue light induces chlorophyll fluorescence, which passes through the filter (Figure 1D). This is a simple method to generate a mask image to separate the plant from the background and analyze it for chlorophyll fluorescence intensity. To collect this mask image, the program triggers a servo motor to rotate the filter in front of the camera. Then, the filter is rotated back out of the image to take the other images. The servo motor is a micro-size hobby remote control model chosen because there exists a robust programming library, making it simple to implement. Servo motors contain a built-in location encoder, allowing for consistent and repeatable movements. The servo motors are a proven design and widely available as well as standardized, allowing for easy replacement. The microcomputer uses relays (although one can use a generic 4-in-1 relay panel, this system utilized HW-316A; EDKG, China) to trigger the different colors of LED strips with the collection of monochrome images under each color of the LED (Figure 1C). A comprehensive list of components used to make the custom system is available in Table 1.

The exposure time of each color during the image acquisition was determined by using a 19 × 25 cm middle-gray card (50% reflection at all wavebands) (WhiBal G7; Eclipse Group LLC/Outdoor Photo Gear, Louisville, KY, USA) as a reference. In summary, we measured the pixel intensity of the gray card image at each color of a LED using ImageJ open-source software (version 1.53k; NIH, Bethesda, MD, USA). The measured intensity was compared to an intensity of 127, which is the midpoint in the 8-bit resolution of the images (0–255) and was the intensity chosen to normalize exposure times. The exposure time of the imaging system was changed to give a pixel intensity of 127 for the gray card image under each LED color. This approach ensures that images taken under different colors of LEDs are directly comparable and accurate measures of reflectance (Figure 2).

## 3. Results

### 3.1. Plant Segmentation via Chlorophyll Fluorescence Imaging

Based on the chlorophyll fluorescence image, our program accurately separated the plant from the background. Since the contrast can be explained by differences in pixel intensity (Figure 3D), an intensity-based thresholding separated the background and plant object, while the higher pixel intensity values corresponded to the plant object and the lower values were derived from the background. Following it, our program automatically optimized a threshold value to generate a mask image. The program identified the two peaks from the left in the histogram of the chlorophyll fluorescence image, which were the background and plant area, respectively. In other words, a minimum value between two peaks was optimal for the intensity-based thresholding. Additionally, despeckling based on pixel size eliminated noise and retained only plant objects in the analysis. The mechanisms are similar in the commercial and in-house systems, though with simplifications. On top of the ability to generate a mask image, there were circumstances in which chlorophyll fluorescence images were highly informative.

### 3.2. Evaluation of NDVI Obtained by Multispectral Imaging

The program calculated canopy NDVI values by deploying the pixel intensities of red and near-infrared images into the NDVI equation on each pixel. With the NDVI value in each pixel and the foreground information, the program created false color canopy NDVI images, while NDVI information was based solely on canopy properties and ignored the background (Figure 4B,D). The leaf chlorosis in Figure 4A matched the lower NDVI color in Figure 4B. The program exported the information in histograms of NDVI proportion and summarized data, including the average and standard deviation over the canopy area, to a comma-separated values file. This differentiation between background and plant material strengthened the relationship between our imaging-based pigment indices and portable meter readings, compared to an imaging-based index that combines canopy and background.

Differences in chlorophyll levels between low and sufficient fertilizer treatments were evident from the average NDVI value of the plants and easily visualized in false-color NDVI images (Figure 4). In the lowest fertilizer treatment, two peaks were observed due to the senescence of an older leaf, which did not occur in plants grown with high fertilizer levels. The NDVI values derived from the image analysis showed an asymptotic relationship with the chlorophyll content index (Figure 5, R^2^ = 0.87, *p* < 0.0001, *n* = 193).

### 3.3. A Low-Cost Imaging System

Upon evaluation of the software for image analysis, we developed a low-cost imaging system. The imaging system takes blue, green, red, and near-infrared pictures under the excitation of each monochromatic LED. With the servo motor-enabled chlorophyll fluorescence capability, the system gained the capability of collecting chlorophyll fluorescence images. The chlorophyll fluorescence image is then used for foregrounding plant objects. A red-green-blue (color) image and an index image were generated for only plant objects. The system also exports the projected canopy size, average, standard deviation, and histogram of the index, such as the NDVI.

Example image sets generated by the low-cost imaging system are shown in Figure 6. Columns A and B represent healthy and chlorotic leaves of hosta (*Hosta plantaginea*) “August lily”, respectively, while showing clear differences in their images. Our system successfully developed an NDVI image only for plants based on foreground using chlorophyll fluorescence images. The average and standard deviation of the NDVI values from healthy and symptomatic leaves were 0.656 ± 0.148 and 0.077 ± 0.077, respectively. Not surprisingly, this is also applicable in indices for other pigments if the spectrum of LEDs used for the system is comparable. For example, the program can generate an image for the normalized difference anthocyanin index (NDAI), an index representing anthocyanin concentration using red and green photos [25]. The NDAI image of two lettuce cultivars with different anthocyanin concentrations from the system (Figure 7) also showed not only a difference in their appearance but also their average and standard deviation (0.482 ± 0.150 and 0.020 ± 0.051 for anthocyanin-rich and anthocyanin-low lettuce cultivars, respectively).

## 4. Discussion

We present the Python code for automated image analysis and a low-cost imaging system that implements this code for automated image collection and analysis [24]. This upload also describes the methodology to replicate the low-cost imaging system, including the Python script, a list of products used in the system, and the three-dimensional computer-aided design file for the filter module and other sundries. This can benefit both researchers and growers who wish to monitor the phenotypic variation in crops objectively. Although there are high-quality imaging systems for various purposes, particularly to monitor spectral reflectance in the wide range of 400 to 1100 nm, such a system is expensive and may not be cost-effective to use in growing areas. Many of these systems do not implement automated image analysis, let alone customizable analysis, or may charge extra to use such software. One of the criteria of a good sensor/system is the simplicity and reproducibility of the method for easy implementation by others. Thus, our approach used open-source software and hardware to be shared with peer researchers and growers interested in using image sensors for their own purposes. We also provide a calibration process for acquiring reliable data using a gray card (having 50% reflection at all wavebands).

The strength of this system is that it can be customized to the user’s needs with simple coding and hardware modifications. To add a single spectrum would require adding a relay and the LED strip, paired with 10–15 lines of code change. For example, facilities in CEA can set up this imaging system without the grow tent, in a pre-existing growth chamber, in an indoor production site with an already known light spectrum, or in a greenhouse at night to have LEDs for image acquisition as the only light source during imaging. Although we only demonstrated NDVI and NDAI imaging as examples, any desired indices regarding plant status can be obtained, limited only by the availability of a light source producing a reliable spectrum for the chosen indices. Another example could be the PRI. In our methodology, we separated the background and plant objects based on chlorophyll fluorescence and intensity-based thresholding. Intensity-based thresholding is a basic but robust approach to separate regions of interest and the background [26]. However, if using a channel of a color image (red, green, blue) or other color models such as hue, saturation, and value, plant images may result in similar pixel intensity between plant objects and background. Supervised machine learning to develop a segmentation model with a training set with labels is commonly used [27,28], but the performance of such models relies on the quality and quantity of training data and its algorithms, which may not be easily accessible to some plant scientists. Our approach to segmenting plant and background is purely based on the optical properties of chlorophyll, abundant in plant species while not in other potential background materials, which fluoresces above 650 nm under blue light excitation and is thus easily captured with a long-pass filter of 650 nm. In addition, we used a low-cost servo motor to place the filter for acquiring the chlorophyll fluorescence image, then removed the filter from the imaging path for the remainder of the time.

**Figure 2:**
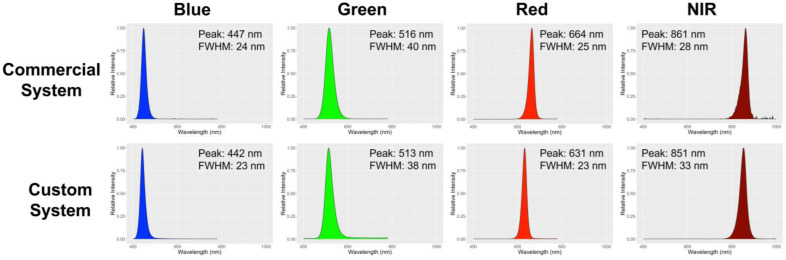
Light spectrum of the commercial and in-house custom imaging systems. The numbers in the figure represent the peak and full-width half maximum (FWHM) of each light spectrum. The color of the peaks matches the representative color of each spectrum.

**Figure 3:**
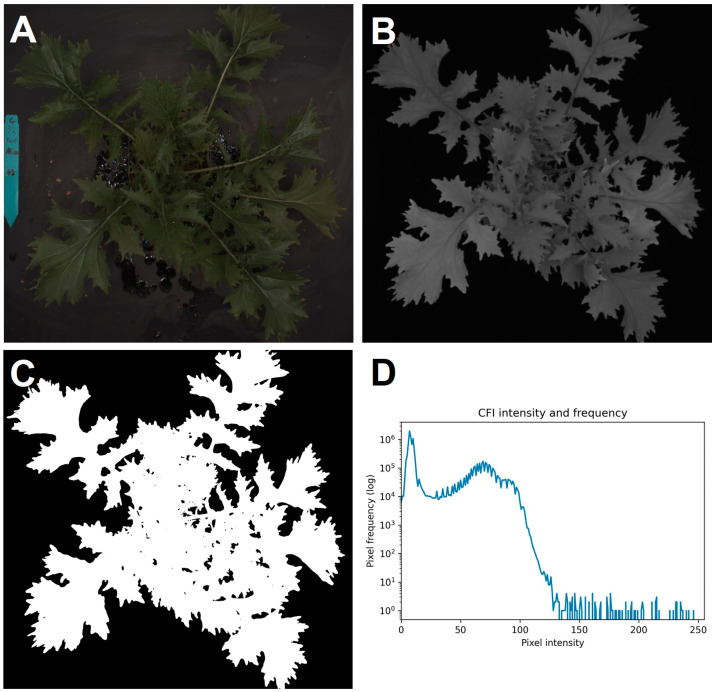
Canopy images of “Mizuna”. Color image (**A**), chlorophyll fluorescence image (**B**), the mask image (**C**), and the pixel intensity distribution of the chlorophyll fluorescence image (**D**).

**Figure 4:**
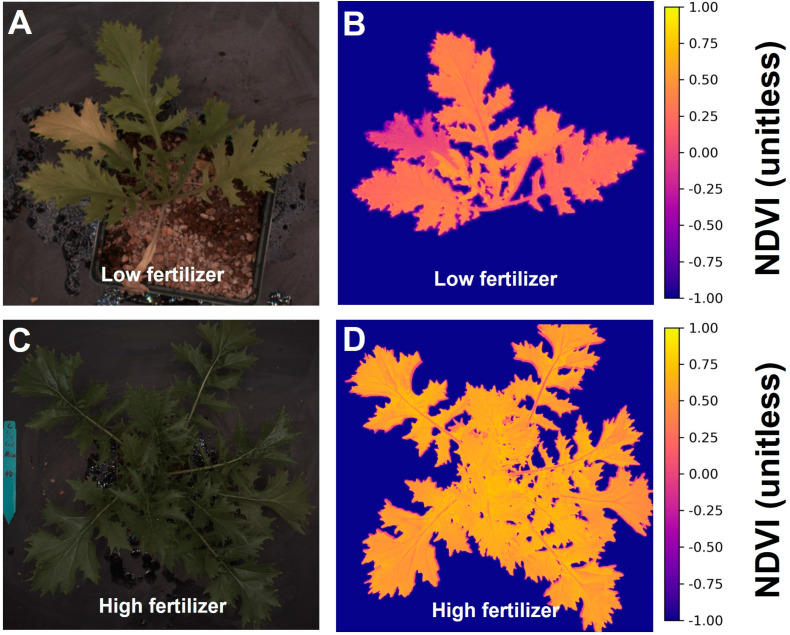
Color images (**A**,**C**) and corresponding normalized difference vegetation index (NDVI) (**B**,**D**) images of “Mizuna” under different fertilizer treatments.

**Figure 5:**
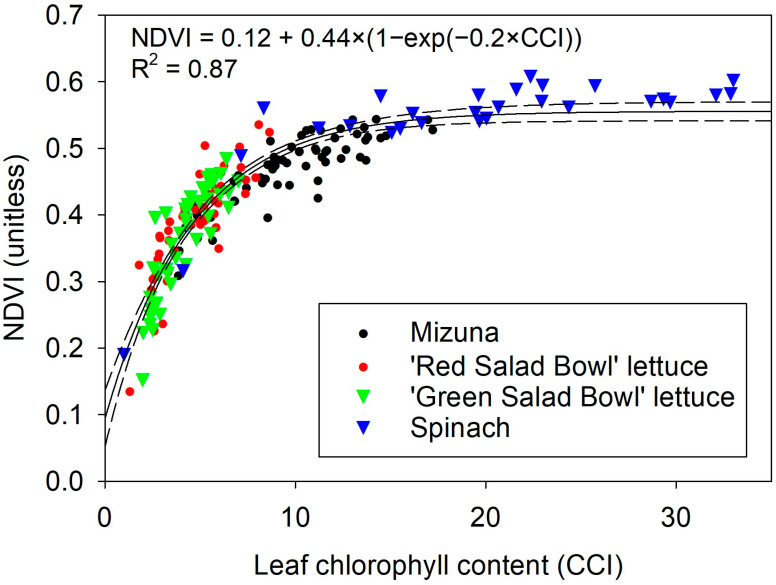
Relationship between normalized difference vegetation index (NDVI) and leaf chlorophyll content readings in four leafy vegetables.

**Figure 6:**
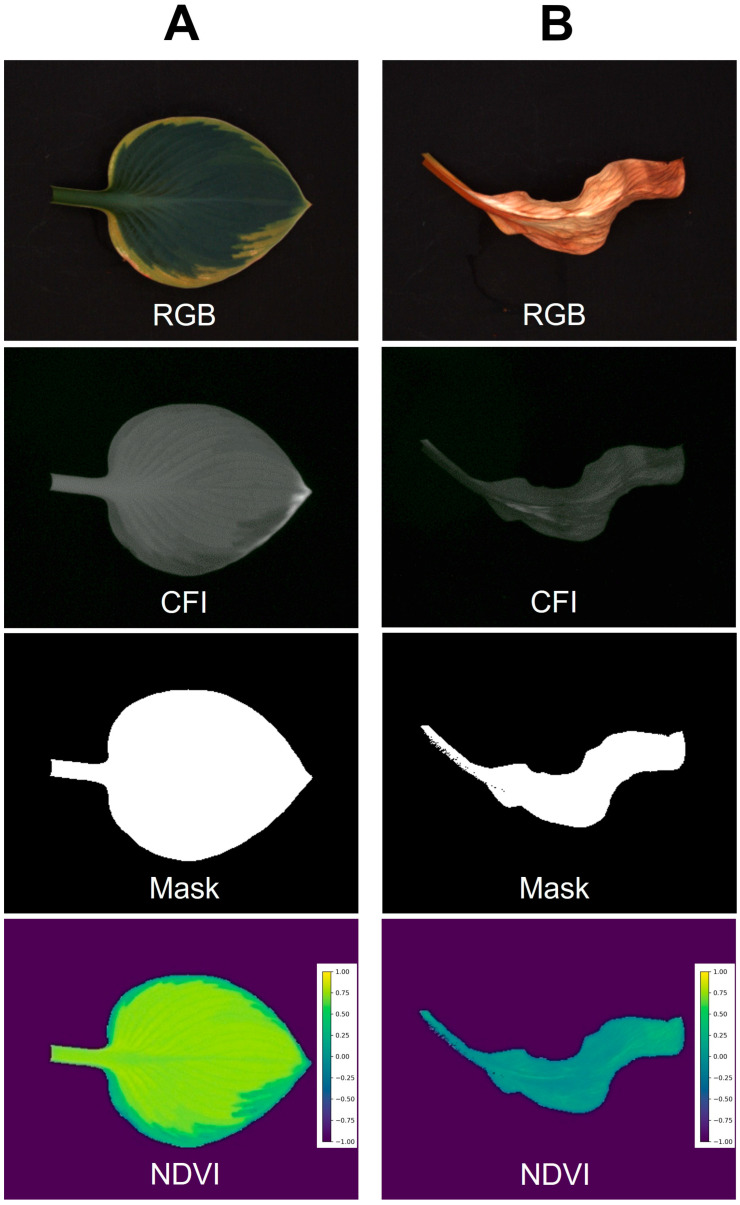
Example images taken by the embedded computer low-cost imaging system. Healthy leaf (**A**) and leaf with chlorosis (**B**) of hosta (*Hosta plantaginea*) “August lily” were used. The color image (RGB), chlorophyll fluorescence image (CFI), mask image for foregrounding, and normalized difference vegetation index (NDVI) are shown.

**Figure 7:**
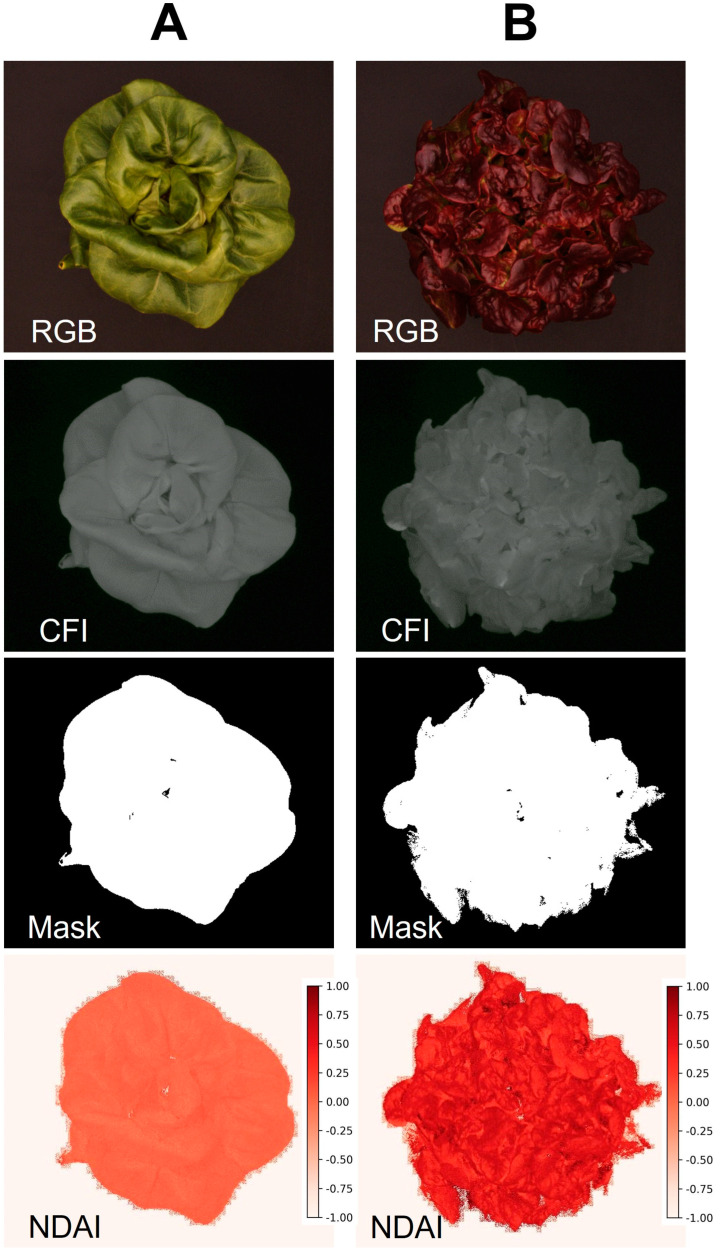
Example images taken by the embedded computer low-cost imaging system. Low anthocyanin lettuce cultivar “Rex” (**A**) and anthocyanin-rich cultivar “Rouxai” (**B**) were used for evaluating the system. The color image (RGB), Chlorophyll fluorescence image (CFI), mask image for foregrounding, and normalized difference anthocyanin index (NDAI) are shown.

Although we only evaluated the image-derived NDVI using the commercial imaging system with chlorophyll content, it is obvious that the result from an embedded computer-driven system will be similar if actinic light during image acquisition is similar. The LED strips we used for our imaging system showed a similar peak and full width at half maximum (Figure 2) as with the commercial imaging system, which supports similarity in the outcomes. Although there was a 31 nm difference in peak between our system and the commercial system in a red light fixture, this difference is acceptable for the NDVI calculation based on the definition of NDVI [4], which defines the red wavelength for NDVI to be 600–700 nm, in which range both imaging systems’ red lights fall. The NDVI equation uses a broad range of red and near-infrared spectra based on the optical properties of chlorophyll, which shows high absorption at most red wavebands and consistently higher reflection between 750 to 900 nm [2,29]. Differences in wavelengths chosen for NDVI calculation generate some variation in NDVI output. The significance of this is unclear, as different wavelengths are used by other companies and researchers [30]. However, we clearly demonstrated that the embedded computer-driven imaging sensor system is feasible for monitoring NDVI at a low cost for research and environmental control in CEA.

The usability of imaging systems can be improved by automated, customized post-processing. Multispectral canopy imaging can extract quantitative information related to anthocyanins and chlorophylls. Chlorophyll fluorescence imaging and its integration with an embedded computer allowed for easy separation of plants from the background and quantification of projected canopy size, and allows for further analysis of pixels that represent plant tissue only. The usability of imaging systems can be improved by automated, customized post-processing. Customizability and automated processing can make multi-spectral imaging available to a wide range of researchers and growers who need such technology but do not otherwise have a starting point to build from.

One of the greatest strengths is the constraint of the imaging area, since the grow tent used is of a fixed size: This makes for a very consistent image collection system allowing for easy comparison of images. This system can be expanded into a greenhouse setting, but the challenge is the need for sole-source lighting, requiring high-quality darkness for consistent images. Acquiring images in the system is limited to a condition of no external light. However, the limitation may not be a problem in CEA. We can simply program the system to take images at night or with no external light condition, which is not difficult.

Some limitations of this image-based phenotyping approach are associated with imaging the plants from above. The images capture the properties of the cell layers near the top of the canopy. If anthocyanins are present on the bottom side of the leaves, the NDAI imaging may not detect them. In addition, the camera can only see one layer of leaves, and the system thus provides no information regarding lower leaves if upper leaves obscure them.

As we discussed, our system may not be suitable for monitoring plant growth in a field or greenhouse during the daytime yet. However, there will be many potential applications of such low-cost imaging systems in other types of CEA, such as vertical farming and/or sole-source lighting conditions. Due to high energy costs in environment control systems, much of the CEA industry has been interested in developing a dynamic environmental control algorithm [31,32]. To do so, a sensor monitoring crop health status will be valuable, while a low-cost imaging system can provide such information in real time. In addition, indoor agriculture often uses artificial light to grow plants, which allows for the acquisition of reliable spectral images for analysis. Additionally, using existing light fixtures such as red or green LEDs can reduce the cost of building this imaging system. While greenhouse or field conditions require more reliable sensors, indoor conditions can be handled by low-cost open-source hardware. Due to the low cost, growers can potentially afford to have multiple setups of such a system to monitor real-time crop growth.

## 5. Conclusions

The usability of imaging systems can be improved by automated, customized post-processing. Multispectral canopy imaging can extract quantitative information related to anthocyanins and chlorophylls. Chlorophyll fluorescence imaging and its integration with an embedded computer allowed for easy separation of plants from the background, enabling further plant tissue analysis only. The simplicity, low cost, and automated processing can make multi-spectral imaging available to a wide range of researchers and growers who need such technology but cannot afford expensive systems.

## Figures and Tables

**Table 1:** Components used to make the custom system, with prices and suppliers, as used, as well as specifications and cost if generic components were used. The totals are ~USD 761 as built and ~USD 385 with generic parts.

Item	Details	Cost as Used	Cost Generic	Manufacturer Information
Raspberry Pi 4b	4 gigabyte random access memory	55	55	Raspberry Pi 4b, The Raspberry Pi Foundation, Cambridge, UK
Light-emitting diode (LED), red	2.5 m	95	10	SimpleColor™ Red, Waveform Lighting, Vancouver, WA, USA
LED, infrared	2.5 m	35	35	850 nm Infrared, Waveform Lighting, Vancouver, WA, USA
LED, blue	2.5 m	95	10	SimpleColor™ Blue, Waveform Lighting, Vancouver, WA, USA
LED, green	2.5 m	95	10	SimpleColor™ Green, Waveform Lighting, Vancouver, WA, USA
Filter	665 nm longpass	81	15	LP665, Midwest Optical Systems, Palatine, IL, USA
4-in-1 relay	12-volt direct current, 4 amp minimum	8	8	HW-316A, EDKG, China
12-volt direct current power supply	5 amp	10	10	12-volt direct current 5 amp, Velain, China
Diffusion plate	opaque/frosted acrylic	20	20	(2MM High Transmittance Supplier Opal Frosted Cast Milky Double-Size PS Diffuser Sheet, Sevenneonlighting, Shenzhen, China
Sample plate	6 mm plywood	20	20	6 mm, 60 × 60 cm plywood, Home Depot, Atlanta, GA, USA
Three-dimensional printed parts		25	25	Custom in-house design, printed in polylactic acid filament (PLA)
64 gigabyte secure digital card		10	10	64 gigabyte micro secure digital card, SanDisk, Milpitas, CA, USA
Grow tent	61 × 61 × 142 cm	100	45	Mylar Reflective Grow Tent for Indoor Hydroponic Growing, Toolots, Cerritos, CA, USA
Servo motor	MG90s	5	5	MG90S, Maxmoral, China
camera	OV9281 Arducam	42	42	OV9281, Arducam, Kowloon, China
Monitor	generic high-definition multimedia interface input	50	50	Philips 22” (55 cm), Philips, Amsterdam, the Netherlands
Keyboard/mouse	Cordless combo	15	15	Keyboard and mouse combo, Rii, Shenzhen, China
Total		761	385	

## Data Availability

Data are contained within the article and Supplementary Materials, and can be provided upon reasonable request.

## Supplementary Materials

The following supporting information can be downloaded at https://zenodo.org/records/10929669 (accessed on 10 July 2024). The link contains Python script for imaging system control and CAD files for system assembly.
